# Radiological staging of rectal cancer in a resource limited setting

**DOI:** 10.1186/s13104-020-05327-4

**Published:** 2020-10-09

**Authors:** Naradha Lokuhetty, Suranjith L. Seneviratne, Fathima Asma Rahman, Thanushka Marapana, Roshan Niloofa, Ishan De Zoysa

**Affiliations:** 1 Department of Surgery, Faculty of Medicine, University of Colombo, Colombo, Sri Lanka; 2 Department of Zoology and Environment Sciences, Faculty of Science, University of Colombo, Colombo, Sri Lanka

**Keywords:** Rectal cancer, Radiological staging, Computed tomography, Magnetic resonance imaging

## Abstract

**Objective:**

Current guidelines on rectal cancer (RC) management recommend pre-operative MRI for loco-regional staging and CT for staging of metastases. This allows appropriate selection of patients for chemo-radiotherapy (CRT). However, MRI is not freely available in many low-income countries. We assessed the status of pre-operative imaging for RC in Sri Lanka and evaluated the performance of CT in RC staging.

**Results:**

A pre-tested interview-administered questionnaire was used to assess the pre-operative use of MRI and CT in RC. CT findings from 37 RC patients were then compared with histopathology findings. Of the 64 surgeons interviewed, 57 (89.1%) did not request an MRI for their RC patients. Reasons cited included limited availability and long waiting times due to competing health needs. A CT was requested by all. In RC, the overall accuracy of CT for T staging was 43.2% and 29.7% of T1–T2 tumours were over-staged as T3. The overall accuracy of CT for regional lymph node staging was 70.3%. In summary, CT alone is not suitable for RC staging in any setting. It leads to over-staging and patients may thus receive unnecessary CRT. Steps must be taken to improve access to pre-operative MRI among Sri Lankan RC patients.

## Introduction

Colorectal cancer (CRC) is the third most common cancer in the world [1]. It’s incidence is increasing in the South Asian region [2, 3, 4]. Rectal cancer (RC) comprises roughly one third of CRC [5]. According to international RC management guidelines, magnetic resonance imaging (MRI) is best for loco-regional staging and computed tomography (CT) for metastatic staging [6, 7]. However, it is often difficult to obtain pre-operative MRI scans for RC patients in many lower-middle income countries including Sri Lanka [8]. CT is cheaper, faster and is more widely available [9, 10]. It is believed that surgeons who operate on RC’s from such countries may not routinely request pre-operative MRI scans.

The National Institute of Clinical Excellence (NICE) 2020 guideline update recommends pre-operative chemo-radiotherapy (CRT) for T3/T4 or any N, M0 RC’s [11]. Neo-adjuvant treatment in RC, down-stages the disease, and reduces overall mortality and disease recurrence [12]. At the National Hospital of Sri Lanka, RC patients with CT stage T3 and T4 receive neo-adjuvant CRT. We assessed the status of pre-operative imaging for RC in Sri Lanka and evaluated the performance of CT in RC staging.

## Main Text

### Methodology

A telephone survey was carried out among 64 surgeons employed in Government hospitals in Sri Lanka who operate on RC patients. A pre-tested questionnaire (see Additional file 1) was used to gather information on each surgeon’s management of RC, including the imaging modalities used. Demographic, clinical, radiological and treatment information of 37 patients with RC managed at the University Surgical Unit were analysed and findings from CT were compared with histopathology. A Toshiba Aquilion Lightening 16 slice CT was used, with scanning done in the arterial, porto-venous and delayed phases. The CT’s were reported by the same specialist radiologists and staged according to the tumour-node-metastasis (TNM) criteria. All CT scans were discussed by the radiologists and surgeons at a weekly meeting prior to the release of a final report. Post-operative specimens were staged according to the American Joint Committee on Cancer Staging (AJCC) 8th TNM model by the Department of Pathology, Faculty of Medicine, University of Colombo [13]. Ethics approval was obtained from the Ethical Review Committee of the National Hospital of Sri Lanka. Simple statistics was carried out to evaluate the accuracy of CT for local tumour (T) and regional lymph node (N) staging of RC.

### Results

Of the 64 surgeons who took part in the survey, 58 (91%) were general surgeons and 6 (9%) were oncology surgeons. Only 7 (10.9%) requested a pre-operative staging MRI on all RC patients, whilst all surgeons requested a CT scan. The reasons given for not ordering an MRI were: limited availability (only six MRI machines are available within the Government Health service in Sri Lanka) and the long waiting times due to the prioritization of MRI’s for other specialities such as Neurology.

Of the 37 patients with RC, 20 (54%) were male. Table 1 compares T staging for RC. The overall accuracy of CT in T staging RC’s was 43.2%. T3 tumours were the most accurately staged at 60%. CT over and under-staged 37.8% (14/37) and 18.9% (7/37) RC’s respectively. 29.7% (11/37) of T1–T2 tumours were over-staged as T3. T2 tumours were most over-staged followed by T1.

**Table 1:** Comparison of T staging for carcinoma involving the rectum

CT Staging	Pathological Staging (n = 37)			
	T1 (n = 7)	T2 (n = 9)	T3 (n = 15)	T4 (n = 6)
T1	3	0	1	0
T2	0	2	2	1
T3	4	7	9	3
T4	0	0	3	2

CT staging of nodal involvement is shown in Table 2. The overall accuracy of CT for regional lymph nodes in RC was 70.3% (26/37). Over and under-staging of lymph nodes by CT was seen in 18.9% (7/37) and 10.8% (4/37) RC’s.

**Table 2:** Comparison of N staging for carcinoma involving the rectum

CT Staging	Pathological Staging (n = 37)		
	N0 (n = 24)	N1 (n = 8)	N2 (n = 5)
N0	18	1	3
N1	5	6	0
N2	1	1	2

### Discussion

We did not find CT to accurately stage RC. T1–T2 tumours were frequently over-staged as T3 by CT. This would result in nearly one-third of patients receiving unnecessary CRT. Over-staging of T1 and T2 RC’s could be a result of peri-rectal fat stranding secondary to rectal inflammation or fibrosis, being interpreted as tumour infiltration [14]. N staging of regional lymph nodes remains a challenge for all modalities. CT had a moderate accuracy of 70.3% for N staging in RC.

At present, MRI and endo-rectal ultra sound (ERUS) are recommended as the primary staging modalities for RC [11, 15]. In resource-limited settings, both these modalities are difficult to access. MRI enables accurate evaluation of tumour extension into the rectal wall and evaluates poor prognostic indicators such as circumferential resection margin (CRM) involvement, extra-mural vascular involvement (EMVI) and a high level of extra-mural spread [16, 17]. Staging of loco-regional lymph nodes are a challenge in RC. MRI is the recommended modality for N staging as it can accurately assess lymph nodes in the mesorectum and pelvic side wall [17].

Surgeons in some developing countries such as Sri Lanka continue to use CT as the primary pre-operative imaging modality for staging RC. The reasons cited include unavailability and long waiting times. Given that 37.8% of RC’s are over-staged, a high proportion of patients may incorrectly receive CRT (and be exposed to its associated complications of enteritis, neutropenia, chronic pain, incontinence and sexual difficulties). It may also add to the healthcare costs in a developing country. In conclusion, CT cannot be relied on for accurately staging RC. The current practice of peri-operative imaging of RC’s in Sri Lanka needs to be changed. A multi-disciplinary team consisting of surgeons, radiologists, oncologists, pathologists, health administrators and policy makers should be convened to find ways to improve access of RC patients to pre-operative staging MRI’s.

## Limitations

A limitation of our study was the relatively low number of RC patients studied.

## Supplementary information

**Additional file 1: Figure S1.** Questionnaire on Imaging in Rectal Cancer – questionnaire aimed at gathering information on the imaging modalities used by Sri – Lankan surgeons for rectal cancer patients.

## Data Availability

All data associated with this manuscript are available on request to the corresponding author.

## Abbreviations

CRC: Colorectal cancer
RC: Rectal cancer
MRI: Magnetic resonance imaging
CT: Computed tomography
NICE: The National Institute of Clinical Excellence
CRT: Chemoradiotherapy
AJCC: American Joint Committee on Cancer Staging
T: Tumour stage
N: Lymph node stage
ERUS: Endo-rectal ultrasound
CRM: Circumferential resection margin
EMVI: Extra-mural vascular invasion
