# Supplementary material for: Radiological staging of rectal cancer in a resource limited setting
Source: BMC Res Notes. 2020 Oct 9;13:479. doi: 10.1186/s13104-020-05327-4 (PMC7547447; doi:10.1186/s13104-020-05327-4)
Supplement: Supplementary file 1 — Additional file 1: Figure S1. Questionnaire on Imaging in Rectal Cancer – questionnaire aimed at gathering information on the imaging modalities used by Sri – Lankan surgeons for rectal cancer patients. [file 13104_2020_5327_MOESM1_ESM.docx]

**Questionnaire on Imaging in Rectal Cancer**

1. What is your surgical specialty?

- General Surgeon
- Gastro-intestinal Surgeon
- Oncological Surgeon
- Colorectal Surgeon

2. Do you treat patients with rectal cancer?

- Yes
- No

If yes,

3. Do you request abdominal CT scans for your rectal cancer patients?

- Yes
- No

4. Do you request pelvic MRI scans for your rectal cancer patients?

- Yes
- No

If no,

5. Do you believe that MRI scans are superior to CT scans for loco-regional staging?

- Yes
- No

If yes,

6. What is the reason for not requesting pelvic MRI scans for rectal cancer patients?

- Limited availability
- Long waiting times
- Other
